# Risk factors associated with early mortality in patients with multiple myeloma who were treated upfront with a novel agents containing regimen

**DOI:** 10.1186/s12885-016-2645-y

**Published:** 2016-08-08

**Authors:** Sung-Hoon Jung, Min-Seok Cho, Hee Kyung Kim, Seok Jin Kim, Kihyun Kim, June-Won Cheong, Soo-Jeoong Kim, Jin Seok Kim, Jae-Sook Ahn, Yeo-Kyeoung Kim, Deok-Hwan Yang, Hyeoung-Joon Kim, Je-Jung Lee, Korean Multiple Myeloma Working Party (KMMWP)

**Affiliations:** 1 Department of Hematology-Oncology, Chonnam National University Hwasun Hospital, 322 Seoyangro, Hwasun, Jeollanamdo 519-763 Republic of Korea; 2 Division of Hematology-Oncology, Department of Medicine, Samsung Medical Center, Sungkyunkwan University School of Medicine, 50 Irwon-dong, Gangnam-gu, Seoul 135-710 Republic of Korea; 3 Division of Hematology, Department of Internal Medicine, Yonsei University College of Medicine, Severance Hospital, Seoul, Republic of Korea

**Keywords:** Early mortality, Comorbidity, Thrombocytopenia, Multiple myeloma

## Abstract

**Background:**

Although the introduction of novel agents improved the survival outcomes in patients with multiple myeloma (MM), some patients died within one year (early mortality, EM) following diagnosis. In this study, we evaluated the EM rate, and investigated the risk factors associated with EM in MM patients.

**Methods:**

Retrospective data from 542 patients who were initially treated with a novel agent-containing regimen were analyzed.

**Results:**

The median overall survival (OS) for the entire cohort was 56.5 months. The median OS in the 2010–2014 group was longer than in the 2002–2009 group (59.2 months vs. 49.1 months, *P* = 0.054). The rate of EM was 13.8 %, and the most common causes of EM were infection and comorbidity. In multivariate analysis, the age-adjusted Charlson comorbidity index (ACCI ≥ 4), low body mass index (BMI < 20 kg/m^2^), thrombocytopenia, and renal failure were significantly associated with EM. The presence of none, 1, or ≥ 2 factors was associated with a 4.1 %, 14.3 %, or 27.4 % risk of EM (*P* < 0.001), respectively. The median OS times were significantly different depending on the presence of factors associated with EM (*P* < 0.001).

**Conclusions:**

In conclusion, the ACCI (≥ 4), low BMI, thrombocytopenia and renal failure were strong predictors for EM in the novel agent era. The results of this study will help to identify patients at high risk for EM, and may be helpful to more accurately predict prognosis of MM patients in the novel-agent era.

## Background

Multiple myeloma (MM) is a clonal B-cell malignancy characterized by aberrant expansion of malignant plasma cells in bone marrow [1]. MM accounts for 1 % of all cancers and more the 10 % of all hematologic malignancies in the United States [2]. In Asian countries, the incidence of MM is lower than that of Western countries, but is increasing rapidly [3]. Treatment options for MM have expanded since the introduction of melphalan in the 1960s. The median survival of MM patients was less than a year prior to the introduction of this alkylating agent, and treatment with melphalan improves survival [4]. In the 1980s, the introduction of high-dose chemotherapy followed by autologous stem cell transplantation (HDT/ASCT) improved the response rate and survival [5–8]. Induction therapy with alkylating agents, anthracyclines and corticosteroid, and HDT/ASCT were the main treatment strategy in MM patients. These treatment paradigms have markedly changed following the introduction of several new agents such as proteasome inhibitor bortezomib, the immunomodulatory drug thalidomide, and its derivative lenalidomide. Treatments with more effective and less toxic agents improved the response rate in relapsed or refractory disease [9, 10]. Additionally, their use during induction resulted in considerable improvement of outcomes and extended the overall survival (OS) times [11, 12]. The treatment improvements in MM patients also affected early mortality (EM), defined as death within 1 year of diagnosis. A recent single center report showed improvement in EM over the last decade in a large –volume, tertiary MM center [13]. However, 10 % of patients still died within 1 year of diagnosis, and the cause and risk factors for EM have not been thoroughly explored in the novel-agent era.

In this study, we evaluated the EM rate and investigated risk factors associated with EM in MM patients initially treated with novel-agent containing regimen.

## Methods

### Patients

This retrospective study analyzed the records of 542 patients with newly diagnosed MM between September 2002 and February 2014 from three institutions in the Republic of Korea. Patients who were initially treated with novel agents such as immunomodulatory drug or proteasome inhibitors were included. Patients diagnosed with monoclonal gammopathy of undetermined significance, asymptomatic MM, and plasma cell leukemia were excluded. Patients who did not receive the induction treatment were also excluded. This study was approved by the Institutional Review Board of Chonnam National University Hwasun Hospital in accordance with the Declaration of Helsinki.

EM was defined as death within one year of diagnosis. Mortality rate and cause at 3, 6, and 12 months following diagnosis was evaluated. Comorbidity score was scored according to the Charlson Comorbidity Index (CCI), as calculated at the time of diagnosis based on the clinical history as well as laboratory and radiologic tests. Age-adjusted Charlson Comorbidity Index (ACCI) was calculated by adding the comorbidity score to the age score, which adds 1 point per decade to ages > 40 years [14]. Body mass index (BMI) was calculated as weight measured in kilograms divided by the square of the height measured in meters (kg/m^2^). Height and weight at diagnosis or prior to first-line chemotherapy were used to calculate BMI. Clinical staging was performed using the International Staging System (ISS). The cytogenetic risk was classified as standard or high risk based on conventional cytogenetic studies or fluorescent *in situ* hybridization. Patients with t(4;14), t(14;16), or, 17p deletion were classified as high risk. Normal cytogenetics and other cytogenetic abnormalities were classified as standard risk. Treatment response was assessed on the first day of each treatment cycle according to the International Myeloma Working Group criteria.

### Statistical analysis

Pearson’s chi-square test for discrete variables and the Mann-Whitney *U* test for continuous variables were used to compare patient characteristics. OS was defined as the period from the date of diagnosis to the date of the last follow-up or death from any cause. OS was evaluated using Kaplan-Meier estimates and compared using log-rank test. Univariate analysis of factors associated with EM was performed with the *χ*^2^ test. Among the factors, those with *P* < 0.05 were selected and included in the multivariate logistic regression analysis. All statistical computations were performed using SPSS ver. 21 (SPSS, Chicago, IL, USA). A *P*-value < 0.05 was considered significant for all analyses.

## Results

### Patient population

The median age of the patients was 63 years (range, 38–86 years) and 43.0 % were ≥ 65 years. A total of 304 patients (56.1 %) were male. The MM type of 296 patients (54.6 %) was Immunoglobulin (Ig) G, and 20.3 % of patients had light chain disease. With regard to the ISS, 133 patients (24.5 %) were stage I, 191 (35.2 %) were stage II, and 211 (38.9 %) were stage III. Of the 542 patients, 188 (34.7 %) diagnosed between 2002 and 2009, and 354 were diagnosed between 2010 and 2014. The clinical characteristics and treatment for these two periods are summarized in Table 1.

**Table 1:** Clinical characteristics

	All patients (*n* = 542)	2002–2009 (*n* = 188)	2010–2014 (*n* = 354)	*P* ^*^
Age, years (range)	63 (31–86)	63.4 (32–85)	62 (31–86)	0.678
≥ 65, *n* (%)	233 (43.0)	86 (45.7)	147 (41.5)	0.363
Male, *n* (%)	304 (56.1)	106 (56.4)	198 (55.9)	0.928
ISS, *n* (%)				
I	133 (24.5)	52 (27.7)	81 (22.9)	0.248
II	191 (35.2)	64 (34.0)	127 (35.9)	0.705
III	211 (38.9)	70 (37.2)	141 (39.8)	0.578
Ig type, n (%)				
IgG	296 (54.6)	111 (59.0)	185 (52.3)	0.173
IgA	113 (20.8)	44 (23.4)	69 (19.5)	0.319
Light chain only	110 (20.3)	31 (16.5)	79 (22.3)	0.116
ECOG PS ≥ 2, *n* (%)	140 (25.8)	54 (28.7)	86 (24.3)	0.304
Median BM plasma cells, %	37.5	34.7	40.0	0.236
Serum albumin, median, g/dL	3.5	3.4	3.5	0.121
Serum β2-microglobulin, median, mg/L	4300	4400	4241	0.709
Primary treatment regimen, *n* (%)				
Thalidomide-based	358 (66.1)	161 (85.6)	197 (55.6)	< 0.001
Bortezomib-based	173 (31.9)	27 (14.4)	146 (41.2)	< 0.001
Lenalidomide-based	10 (1.8)	0	10 (2.8)	0.018
Carfilzomib-based	1 (0.2)	0	1 (0.3)	1.000
Performance of ASCT, *n* (%)	233 (43.0)	71 (37.8)	162 (45.8)	0.083

Abbreviations: *N* number, *ISS* International Staging System, *Ig* immunoglobulin, *ECOG* Eastern Cooperative Oncology Group, *PS* performance status, *BM* bone marrow, *ASCT* autologous stem cell transplantation

^*^Comparison between the 2002–2009 and 2010–2014 cohorts

A total of 215 patients (39.7 %) had at least one comorbidity at the time of diagnosis, and 45.1 % of elderly patients (≥ 65 years) had a concurrent comorbidity. The median ACCI score was three for the entire group (range, 0–9). The median BMI was 23.3 kg/m^2^ (range, 13.1–59.2) at the time of diagnosis. Underweight patients (< 18.5 kg/m^2^) accounted for 13 (2.3 %), and 16 patients (2.9 %) were obese (≥ 30 kg/m^2^).

All patients were treated with a regimen containing a novel agent after the initial diagnosis. A total of 358 patients (66.1 %) received a thalidomide-based regimen as the first-line treatment, such as cyclophosphamide, thalidomide, and dexamethasone (CTD), or melphalan, prednisolone, and thalidomide (MPT), or thalidomide alone or other combinations. A total of 173 patients (31.9 %) were treated with a bortezomib–containing regimen such as bortezomib, melphalan, and prednisolone (VMP), or bortezomib, cyclophosphamide, and dexamethasone (VCD), or bortezomib and other combinations. Ten patients (1.8 %) were treated with lenalidomide and low-dose dexamethasone. One patient received the carfilzomib, melphalan, and prednisolone regimen. Because front-line treatment with bortezomib was not covered by health insurance in Korea at 2002–2009, the majority of patients during this time were treated with a thalidomide-based regimen as induction therapy. In elderly patients, majority of patients (82.6 %) were treated with a thalidomide-based regimen at 2002–2009, but 85.0 % of patients were treated with a bortezomib-based regimen at 2010–2014 by health insurance. In addition, lenalidomide was not used the front-line treatment by 2009, and eight elderly patients (5.4 %) received the lenalidomide-based regimens as the front-line therapy at 2010–2014.

### Survival outcomes and factors associated with OS

**Fig. 1:**
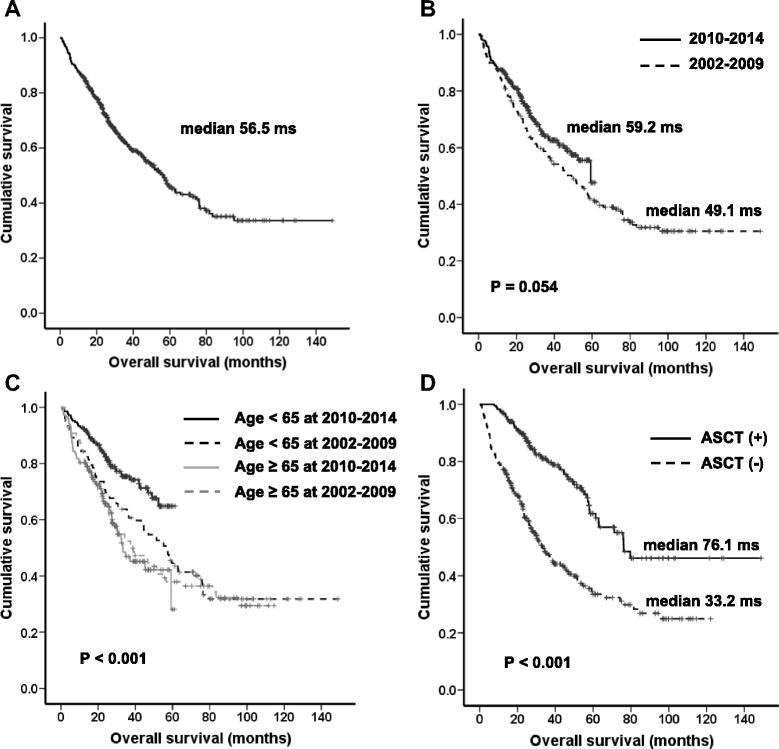
**a** Kaplan-Meier survival curves for overall survival (OS) in all patients. **b** OS according to age. **c** OS according to the period of diagnosis. **d** OS comparison between patients receiving an autologous stem cell transplantation (ASCT) versus those who did not receive ASCT0

Over a median follow up of 34.6 months, the median OS was 56.5 months (95 % CI 48.6–64.4, Fig. 1a). The median OS for the 2010–2014 group was longer compared with the 2002–2009 group (59.2 months vs. 49.1 months, *P* = 0.054, Fig. 1b). This improved OS was primarily seen in patients under 65 years of age (not reached vs. 56.8 months, *P* = 0.009). There was no significant difference in OS according to the diagnosis period in patients over the 65 years of age (33.2 months vs. 37.9 months, *P* = 0.805, Fig. 1c). Among the entire group, a total of 233 patients (43.0 %) underwent HDT/ASCT, and 74.1 % of patients under 65 years of age received HDT/ASCT. The median time to HDT/ASCT from diagnosis was 5.9 months (3.1–45.4 months). Patients who received HDT/ASCT had significantly longer OS compared with those who did not undergo HDT/ASCT (76.1 months vs. 33.2 months, *P* < 0.001, Fig. 1d). In young patients (< 65 years), the median OS for patients receiving HDT/ASCT was significantly longer than those who did not undergo HDT/ASCT (76.1 months vs. 23.5 months, *P* < 0.001).

**Fig. 2:**
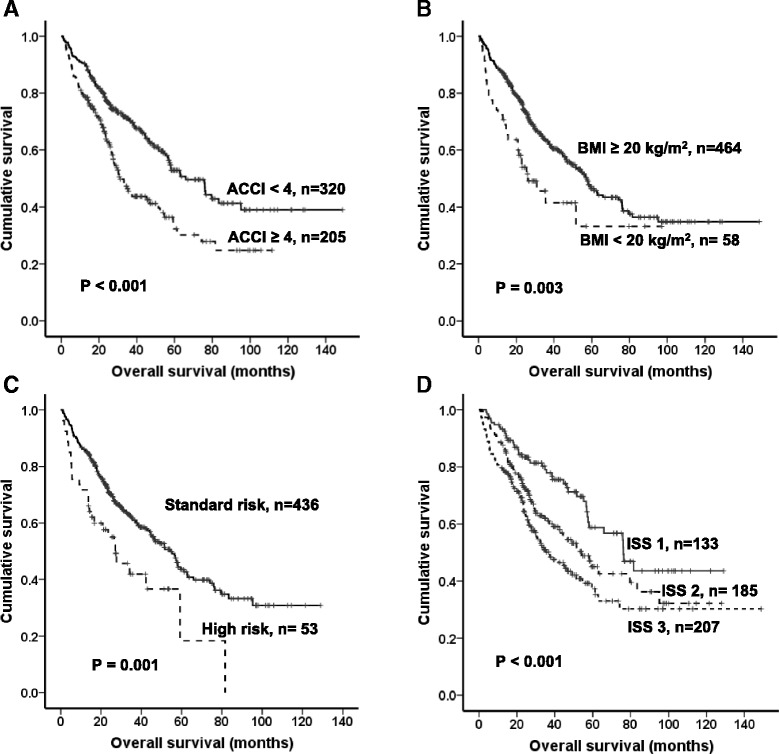
Kaplan-Meier survival curves for overall survival (OS) according to **a** age-adjusted Charlson Comorbidity Index (ACCI) 4, **b** body mass index (BMI) 20 kg/m^2^, **c** cytogenetic risk, and **d** international staging system (ISS)

We evaluated the factors associated with OS in 525 patients, excluding patients who did not have certain laboratory test at the initial diagnosis. Cox multivariate analysis showed: ACCI ≥ 4 [hazard ratio (HR), 1.782; 95 % CI, 1.227–2.587; *P* = 0.002], BMI < 20 kg/m^2^ (HR 1.780, 95 % CI 1.177–2.693, *P* = 0.006), ECOG performance status (PS) ≥ 2 (HR 1.468, 95 % CI 1.064–2.025, *P* = 0.019), and high risk cytogenetics (HR 1.625, 95 % CI 1.063–2.486, *P* = 0.025). ISS was prognostic for OS in univariate analysis but not in multivariate analysis (Fig. 2).

### Characteristics of EM

**Fig. 3:**
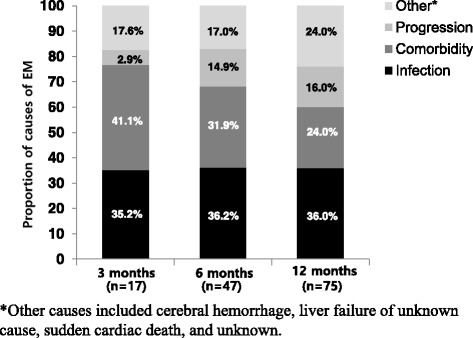
Etiologies of EM

Of the 542 patients, 75 (13.8 %) died within 12 months of diagnosis, 3.1 % within 3 months, and 8.6 % within 6 months. The EM rate in the 2010–2014 group was lower (12.7 % vs. 15.9 %, *P* = 0.356). The causes of mortality at 3, 6 and 12 months are summarized in Fig. 3. The major cause of EM was infection and comorbidity. The most common form of infection was septic shock with pneumonia, and the rate of death from infection remained constant for 12 months (35.2 % at 3 months, 36.2 % at 12 months). Pathogens and types of infection summarized in Table 2. Mortality from comorbidity (41.1 %) was higher than those from infection at 3 months, but it has decreased gradually to 24.0 % at 12 months. Comorbidities associated with EM were cardiac disease (55.5 %), renal disease (22.2 %), hepatic disease (5.5 %), chronic lung disease (5.5 %), and solid tumor (11.1 %). Death from disease progression was low for the 3 months and then increased gradually.

**Table 2:** Infectious complications of early mortality

	Number (%)
MDI	
Pneumonia	
Streptococcus pneumoniae	2 (7.4 %)
Pseudomonas aeruginosa	2 (7.4 %)
Klebsiella pneumoniae	1 (3.7 %)
Escherichia coli	1 (3.7 %)
Influenza A	1 (3.7 %)
Pneumocystis jiroveci	1 (3.7 %)
Meningoencephalitis	
Streptococcus pneumoniae	1 (3.7 %)
CDI	
Pneumonia	12 (44.4 %)
Unknown	6 (22.2 %)

*MDI* microbiologically documented infection, *CDI* clinical documented infection

### Factors associated with EM

**Fig. 4:**
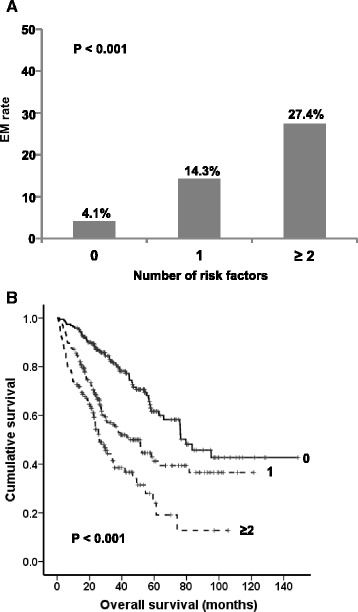
**a** EM rate and **b** OS according to number of risk factors

Univariate analysis showed that age > 70 years, BMI < 20 kg/m^2^, ECOG PS ≥ 2, ACCI ≥ 4, high lactate dehydrogenase, low absolute lymphocyte count (≤ 1.1 × 10^9^/L), platelets < 100 × 10^9^/L, serum creatinine ≥ 2 mg/dL, serum albumin < 3.5 g/dL, and serum β2-microglobulin > 5500 mg/L were significantly associated with EM. These variables were examined using multivariate analysis, which identified ACCI ≥ 4, BMI < 20 kg/m^2^, platelets < 100 × 10^9^/L, and serum creatinine ≥ 2 mg/dL as factors that independently predict EM (Table 3). The presence of 0, 1, or ≥ 2 factors was associated with a 4.1 %, 14.3 %, or 27.4 % risk of EM (*P* < 0.001, Fig. 4a), respectively. The median OS was significantly different depending on the presence of factors associated with EM (*P* < 0.001, Fig. 4b).

**Table 3:** Univariate and multivariate analysis of risk factors associated with EM (*n* = 525)

Variables	Univariate analysis		Multivariate analysis	
	HR (95 % CI)	*P*	HR (95 % CI)	*P*
Age > 70 years	2.13 (1.23–3.70)	0.007		
Female	0.70 (0.42–1.17)	0.183		
Body mass index < 20 kg/m^2^	2.72 (1.43–5.15)	0.002	2.26 (1.12–4.56)	0.022
ECOG PS ≥ 2	1.82 (1.08–3.06)	0.024		
ACCI ≥ 4	2.56 (1.55–4.24)	< 0.001	2.23 (1.14–4.36)	0.019
LDH > 1 x ULN	1.98 (1.14–3.46)	0.015		
ALC ≤ 1.1 × 10^9^/L	2.26 (1.28–4.00)	0.005		
Hemoglobin < 10 g/dL	1.36 (0.81–2.27)	0.233		
Platelet < 100 × 10^9^/L	2.66 (1.59–4.42)	< 0.001	2.21 (1.27–3.84)	0.005
Serum creatinine ≥ 2 mg/dL	3.52 (2.07–6.01)	< 0.001	2.37 (1.18–4.76)	0.015
Serum albumin < 3.5 g/dL	1.82 (1.10–3.01)	0.020		
Serum β2-microglobulin > 5500 mg/L	2.83 (1.71–4.68)	< 0.001		

Abbreviations: *EM* early mortality, *ECOG* Eastern Cooperative Oncology Group, *PS* performance status, *ACCI* age-adjusted Charlson comorbidity index, *CCI* Charlson comorbidity index, *LDH* lactate dehydrogenase, *ULN* upper limit of normal vaule, *ALC* absolute lymphocyte count

## Discussion

The introduction of novel agents with new therapeutic mechanisms has changed the paradigm of MM therapy, and considerably improved outcomes in patients with this disease. Recent studies have demonstrated that improved survival has been sustained following the introduction of novel agents in elderly patients as well as younger patients [13, 15]. These survival data mainly come from studies based in Western countries, while data for Asian patients are rare. In this study, Korean MM patients also exhibited improved survival following the introduction of novel agents, but improved survival was limited to younger patients. The lack of improved survival in elderly patients may be associated with health insurance limitations in Korea. The use of synthetic derivatives of thalidomide, such as lenalidomide and pomalidomide, were not approved in Korea during 2002–2014.

There was a 13.8 % rate of EM in patients initially treated with novel-agent containing regimen, and the main causes of EM were infection and comorbidity. Infection was reported as a major cause of morbidity and a leading cause of death in patients with MM [16, 17]. MM patients are predisposed to infection because of immune dysfunction, placement of vascular catheters, and impaired mucosal integrity due to the effects of chemotherapy and radiotherapy [18, 19]. In a recent large population-based study of over 9000 MM patients in the period 1988–2007, the risk of infections and infection-related death is significantly increased in MM patients compared to controls, and the incidence of infection was highest within the first year following diagnosis. Furthermore, the risk of infection has increased in the recent decades [20]. In our study, the rate of death from infection was constant within a 12 month period. These results suggest that infection is still a major problem for MM patients treated with novel-agent containing regimen, and the management of early infection throughout the disease course was important to improve survival.

Another goal of this study was to identify factors associated with EM after induction treatment. Because comorbidities present at diagnosis were identified as a major cause of EM in this study, we evaluated the prognostic value of ACCI. The CCI is a statistically validated tool that assigns different weights to patients’ comorbidities to predict mortality, and can be adjusted to the patients age [14]. CCI has been demonstrated to affect survival outcomes for various types of cancer, including hematologic malignancies [21–23]. In patients with MM, several reports showed that comorbidity score was associated with diminished survival outcomes [24, 25]. Comorbidities such as renal impairment, impaired lung function, and poor PS were prognostic for poor OS in MM patients. In multivariate analysis, high ACCI (≥ 4) was significantly associated with EM. In addition, low BMI (< 20 kg/m^2^) was significantly associated with EM, and 27.1 % of patients with low BMI died within 12 months of diagnosis. We previously reported that a low BMI (< 20 kg/m^2^) at the time of diagnosis was associated with poor survival [26]. Low BMI may reflect involuntary weight loss caused principally by cancer-associated cachexia [27]. High ACCI and low BMI may be associated with reduced physical function, poorer tolerance of treatment, and increased toxicity of chemotherapy. Therefore, ACCI and BMI are considered as an important host factors to stratify the risk of myeloma and decide treatment options.

The prognostic role of thrombocytopenia is less understood in patients with MM. Several reports explored the prognostic role of thrombocytopenia in MM patients with renal failure. One study showed that thrombocytopenia (< 130 × 10^9^/L) was related to a poor prognosis (HR 2.150, 95 % CI 1.167–3.962, *P* = 0.014) [28], whereas another study reported no association (HR 1.52, 95 % CI 0.875–2.65, *P* = 0.136) [29]. In the current study, thrombocytopenia present at initial diagnosis was a strong predictor for EM. A recent study suggested that thrombocytopenia (< 200 × 10^9^/L) was associated with EM, but this was not confirmed by multivariate analysis [13]. Further study is needed to ascertain whether thrombocytopenia has a prognostic role, as well as to determine the appropriate threshold.

This study has some limitations. Validation is an important step in developing a prognostic model. However, the risk factors used in this study were not validated in a separate analysis. The lack of validation step of risk factors can be a limitation of this analysis. Additionally, number of all patients was numerous but number of patients in each group was not very large.

## Conclusion

In conclusion, the rate of MM patients in novel-agent era was 13.8 %. Major causes of EM were infection and comorbidity. The ACCI (≥ 4), BMI (< 20 kg/m^2^), thrombocytopenia, and renal failure were significantly associated with EM. Median OS times were significantly different depending on the presence of risk factors associated with EM. The results of this study will help to identify patients at high risk for EM, and may be helpful to more accurately predict prognosis of MM patients in the novel-agent era.

## Abbreviations

ACCI, age-adjusted Charlson comorbidity index; BMI, body mass index; CTD, cyclophosphamide, thalidomide and dexamethasone; EM, early mortality; HDT/ASCT, high-dose chemotherapy followed by autologous stem cell transplantation; HR, harzard ratio; ISS, International Staging System; MM, multiple myeloma; MPT, melphalan, prednisolone, and thalidomide; OS, overall survival; PS, performance status; VCD, bortezomib, cyclophosphamide, and dexamethasone; VMP, bortezomib, melphalan, and prednisolone

## References

[1] Kyle RA, Rajkumar SV (2004). Multiple myeloma. N Engl J Med.

[2] Siegel RL, Miller KD, Jemal A (2015). Cancer Statistics, 2015. CA Cancer J Clin.

[3] Kim K, Lee JH, Kim JS, Min CK, Yoon SS, Shimizu K, Chou T, Kosugi H, Suzuki K, Chen W (2014). Clinical profiles of multiple myeloma in Asia-An Asian Myeloma Network study. Am J Hematol.

[4] Osgood EE (1960). The survival time of patients with plasmocytic myeloma. Cancer Chemother Rep 1.

[5] Barlogie B, Smith L, Alexanian R (1984). Effective treatment of advanced multiple myeloma refractory to alkylating agents. N Engl J Med.

[6] Attal M, Harousseau JL, Stoppa AM, Sotto JJ, Fuzibet JG, Rossi JF, Casassus P, Maisonneuve H, Facon T, Ifrah N (1996). A prospective, randomized trial of autologous bone marrow transplantation and chemotherapy in multiple myeloma. Intergroupe Francais du Myelome. N Engl J Med.

[7] Child JA, Morgan GJ, Davies FE, Owen RG, Bell SE, Hawkins K, Brown J, Drayson MT, Selby PJ, Medical Research Council Adult Leukaemia Working P (2003). High-dose chemotherapy with hematopoietic stem-cell rescue for multiple myeloma. N Engl J Med.

[8] Koreth J, Cutler CS, Djulbegovic B, Behl R, Schlossman RL, Munshi NC, Richardson PG, Anderson KC, Soiffer RJ, Alyea EP (2007). High-dose therapy with single autologous transplantation versus chemotherapy for newly diagnosed multiple myeloma: A systematic review and meta-analysis of randomized controlled trials. Biol Blood Marrow Transplant.

[9] Singhal S, Mehta J, Desikan R, Ayers D, Roberson P, Eddlemon P, Munshi N, Anaissie E, Wilson C, Dhodapkar M (1999). Antitumor activity of thalidomide in refractory multiple myeloma. N Engl J Med.

[10] Richardson PG, Sonneveld P, Schuster MW, Irwin D, Stadtmauer EA, Facon T, Harousseau JL, Ben-Yehuda D, Lonial S, Goldschmidt H (2005). Bortezomib or high-dose dexamethasone for relapsed multiple myeloma. N Engl J Med.

[11] Kristinsson SY, Landgren O, Dickman PW, Derolf AR, Bjorkholm M (2007). Patterns of survival in multiple myeloma: a population-based study of patients diagnosed in Sweden from 1973 to 2003. J Clin Oncol.

[12] Kumar SK, Rajkumar SV, Dispenzieri A, Lacy MQ, Hayman SR, Buadi FK, Zeldenrust SR, Dingli D, Russell SJ, Lust JA (2008). Improved survival in multiple myeloma and the impact of novel therapies. Blood.

[13] Kumar SK, Dispenzieri A, Lacy MQ, Gertz MA, Buadi FK, Pandey S, Kapoor P, Dingli D, Hayman SR, Leung N (2014). Continued improvement in survival in multiple myeloma: changes in early mortality and outcomes in older patients. Leukemia.

[14] Charlson M, Szatrowski TP, Peterson J, Gold J (1994). Validation of a combined comorbidity index. J Clin Epidemiol.

[15] Kristinsson SY, Anderson WF, Landgren O (2014). Improved long-term survival in multiple myeloma up to the age of 80 years. Leukemia.

[16] Perri RT, Hebbel RP, Oken MM (1981). Influence of treatment and response status on infection risk in multiple myeloma. Am J Med.

[17] Augustson BM, Begum G, Dunn JA, Barth NJ, Davies F, Morgan G, Behrens J, Smith A, Child JA, Drayson MT (2005). Early mortality after diagnosis of multiple myeloma: analysis of patients entered onto the United kingdom Medical Research Council trials between 1980 and 2002--Medical Research Council Adult Leukaemia Working Party. J Clin Oncol.

[18] Luque R, Brieva JA, Moreno A, Manzanal A, Escribano L, Villarrubia J, Velasco JL, Lopez-Jimenez J, Cervero C, Otero MJ (1998). Normal and clonal B lineage cells can be distinguished by their differential expression of B cell antigens and adhesion molecules in peripheral blood from multiple myeloma (MM) patients--diagnostic and clinical implications. Clin Exp Immunol.

[19] Nucci M, Anaissie E (2009). Infections in patients with multiple myeloma in the era of high-dose therapy and novel agents. Clin Infect Dis.

[20] Blimark C, Holmberg E, Mellqvist UH, Landgren O, Bjorkholm M, Hultcrantz M, Kjellander C, Turesson I, Kristinsson SY (2015). Multiple myeloma and infections: a population-based study on 9253 multiple myeloma patients. Haematologica.

[21] Jehn CF, Boning L, Kroning H, Pezzutto A, Luftner D (2014). Influence of comorbidity, age and performance status on treatment efficacy and safety of cetuximab plus irinotecan in irinotecan-refractory elderly patients with metastatic colorectal cancer. Eur J Cancer.

[22] Sperr WR, Wimazal F, Kundi M, Baumgartner C, Nosslinger T, Makrai A, Stauder R, Krieger O, Pfeilstocker M, Valent P (2010). Comorbidity as prognostic variable in MDS: comparative evaluation of the HCT-CI and CCI in a core dataset of 419 patients of the Austrian MDS Study Group. Ann Oncol.

[23] Etienne A, Esterni B, Charbonnier A, Mozziconacci MJ, Arnoulet C, Coso D, Puig B, Gastaut JA, Maraninchi D, Vey N (2007). Comorbidity is an independent predictor of complete remission in elderly patients receiving induction chemotherapy for acute myeloid leukemia. Cancer.

[24] Kim SM, Kim MJ, Jung HA, Kim K, Kim SJ, Jang JH, Kim WS, Jung CW (2014). Comparison of the Freiburg and Charlson comorbidity indices in predicting overall survival in elderly patients with newly diagnosed multiple myeloma. BioMed Res Int.

[25] Kleber M, Ihorst G, Terhorst M, Koch B, Deschler B, Wasch R, Engelhardt M (2011). Comorbidity as a prognostic variable in multiple myeloma: comparative evaluation of common comorbidity scores and use of a novel MM-comorbidity score. Blood Cancer J.

[26] Jung SH, Yang DH, Ahn JS, Lee SS, Ahn SY, Kim YK, Kim HJ, Lee JJ (2014). Decreased body mass index is associated with poor prognosis in patients with multiple myeloma. Ann Hematol.

[27] Skipworth RJ, Stewart GD, Dejong CH, Preston T, Fearon KC (2007). Pathophysiology of cancer cachexia: much more than host-tumour interaction?. Clin Nutr.

[28] Eleftherakis-Papapiakovou E, Kastritis E, Roussou M, Gkotzamanidou M, Grapsa I, Psimenou E, Nikitas N, Terpos E, Dimopoulos MA (2011). Renal impairment is not an independent adverse prognostic factor in patients with multiple myeloma treated upfront with novel agent-based regimens. Leuk Lymphoma.

[29] Dimopoulos MA, Delimpasi S, Katodritou E, Vassou A, Kyrtsonis MC, Repousis P, Kartasis Z, Parcharidou A, Michael M, Michalis E (2014). Significant improvement in the survival of patients with multiple myeloma presenting with severe renal impairment after the introduction of novel agents. Ann Oncol.

